# Practical Observations on the Causes and Treatment of Curvatures of the Spine

**Published:** 1839-04-01

**Authors:** 


### Practical Observations on the Causes and Treatment of Curvatures of the Spine. By *Samuel Hare*. 1838.

An analysis of this book, addressed as it is more to the public than to the profession, would be misplaced; but we will glance at one or two of the opinions of the author.

After speaking of curvature of the spine in general, he treats of the various forms in which it may shew itself, viz. lateral curvature, angular curvature or projection, excurvation, and incurvation. We notice as a defect, that he has not sufficiently distinguished the essential differences between these, but has treated them as mere varieties of the same disease, distinguished from each other rather by the difference of the curve, than by any essential difference in the nature of the affections producing them. Thus, *lateral curvature*, from the tenor of his observations, would seem equally to be a result of disease of the spine, properly so called, as is angular projection: certainly, he does mention that the latter generally arises from caries, but he does not sufficiently dwell upon the difference of the nature of the former; but rather, by making general observations, which may be applied to either, would lead a reader (and especially an unprofessional reader) to infer, that he considers them, not as distinct diseases, but as varieties of the same; nay, after a careful perusal, we are unable to say, that the author does not consider setons, blisters, &c. as applicable to one of these as to the other. Indeed, the term *diseased spine*, applied to the simply *distorted* spine from lateral curvature, we consider as objectionable, confounding it, as it does, with the very different affection usually known by that name, and thus giving rise to erroneous views of its nature and treatment.

Mr. Hare considers, that lateral curvature arises from a softened state of the bones, produced by a prior state of deranged health, and acted upon by undue pressure, whereas it is well known, that at first, at least, the bones are in their natural condition: he lays, also, too great stress upon the pushing or compressing effects of the *stays* upon the spinal column and upper extremities, as a *direct* means of producing the distortion: not that this detestable instrument of torture can be well too-much blamed or criticised, but our condemnations and criticisms are forcible and effectual only in proportion as they are justly and judiciously directed: and thus the chief hurtfulness of this article of dress arises, not from the immediate effects of the pressure it itself exerts upon the spinal column, &c., but, from the enfeebled state of the muscular supports of the spine, (the natural stays of the body,) it engenders, by the inaction it obliges. Without pretending to be much versed in the history of the toilet, we think we may assert, that the laces of our modern belles are not drawn tighter than those of their predecessors were wont to be; but their being less engaged in domestic and active occupations, and more and more sacrificed to the modern mania for book-learning and accomplishments, entailing upon them nearly continual confinement, and most irksome and unvaried positions, will serve to account for that alarming increase of twisted spines observed of late years.

The author's chapter on treatment is but a meagre affair, for besides some obvious directions, as to the attention required for the general health, he seems to confine himself to the recommendation of the use of his "apparatus," which he declares to be applicable to the treatment of all kinds of curvatures, and even to the relief of cases of spinal irritation; but how long this apparatus requires generally to be continued—how its use should be modified for some cases, or forbidden for others (if any), he informs us not: and indeed we must infer that, for its successful application in cases usually considered as requiring very varying treatment, the personal inspection of its inventor would be necessary. We had intended transcribing his description of this apparatus, but for the mere purposes of criticism it is too long, and we will only advert to its nature, and the objects he has in view in employing it. It consists of an inclined plane, having pullies arranged in different directions, to which are attached weights, and straps for making extension between the head, axilla, and ankles, and sometimes from one or both shoulders, while compresses may be so adjusted, as to make the desirable degree of pressure on the projecting spine, sternum, hip, or side, as the case may be. The author thus sums up the objects he has in view: the italics are our own.

" 1st. By means of the *inclined plane and extension, to bring* the *bony structure* of the body into as near a form of symmetry as may be, and, of course, to *keep* it in that state.

" 2nd. By medicinal treatment, to improve the general health, *forward the deposition of osseous matter in the bones,* and assist Nature in establishing the healthy functions of each organ.

" 3d. By friction and shampooing, *as a substitute for exercise,* (he says elsewhere 'confinement absolutely necessary,') or in some cases by hand-swings, and other gymnastic exercises, *compatible with the first* object of treatment, to develop the muscular structure." 127.

Few, indeed, would be the cases of lateral curvature in which we could consent to condemn a patient to this stretching and pushing practice, considering it as we do, not only as unscientific and injurious in its nature, but also as negatively hurtful, by consuming much time, (many hours daily being passed upon the plane,) which might be employed in well-directed exercises. Even supposing, that by these means the spinal column could be drawn or pushed into its natural position, yet, afterwards, in order to retain it there, powerful muscular supports are as necessary as ever, and in the enfeebled state of the system, found in these cases, these are only to be generated by judicious, varied, regular, but not exhausting exercises, followed by simple horizontal repose. We doubt whether much good can ever be accomplished by machinery after the deformity has become fully formed, while, for the prevention of its progress in the early stage, there can be no question, that to such means we must not resort, but seek by free exercise, and an absence of fatiguing or injurious positions, to avert the threatened mischief. The reader will find some excellent observations upon this subject, in one of Mr. Lawrence's lectures.*

It can never be urged too often upon our medical brethren to use their utmost influence in their respective walks in life, to *prevent,* as far as in them lies, the farther increase of this terrible infliction upon modern refinement. Much may be done by their advice, judiciously given, and *strenuously urged:* often by it they may procure a degree of immunity from corporeal restraint in early life, which will materially aid the frame in resisting afterwards the tyrannic grasp of fashion. The following note of the author is injudicious:—

" It is a mistaken opinion, and attended with some degree of injustice, to attribute the prevailing cause of this extensive and distressing evil to the customs and discipline adopted at public (?) schools: the cause is in operation, and the disease generally commences long before the period at which children are accustomed to leave the parental roof. A remark of this nature seems the more necessary, as the conductors of ladies' seminaries have often a degree of censure cast upon them, which they by no means deserve." 66.

Viewing the affection, as we do, as generally resulting from deficient

---

* Medical Gazette, Vol. VI. p. 612.

muscular exertion, too close confinement, and the maintenance of irksome postures, we are convinced that it very often originates, and is always aggravated, by the discipline observed at ladies' schools, than which, one more destructive to health we cannot imagine: true it is, that parents are ultimately the cause of this, by cruelly and absurdly requiring so much to be taught to their children. That some governesses wish to emancipate themselves from these prejudices we know, but that the great bulk encourage them everybody knows: in fact, the present race of governesses, from their total ignorance of the first principles which should direct their physical education, are totally incapacitated for taking charge of our female youth. The crying want in this country is, *instruction for the instructors.*

An observation is requisite upon the proposal for treating the *angular curvature or projection.*

"The necessity of early attention to the treatment of angular deformity will be sufficiently evident from the consideration of the state of the vertebræ: after the softened portion has suffered compression for some time, the bodies become absorbed, ossific matter is thrown out, which unites and consolidates the approximated surfaces of the bones in the position in which they happen to be placed: hence the absolute necessity of attending to the state and position of the spine in the earliest stages of the disease. If the ossific matter be allowed to accumulate upon the vertebræ, while the column is in a distorted form, the process of restoration will evidently be difficult, and altogether inefficient: but if it receive early and requisite attention while in a soft state—if it can be made straight, and kept so sufficiently long for the ossific matter to deposit itself in the vacant space, an anchylosis free from deformity, or nearly so, will be the result." 90.

We think our author will find few medical men who will be willing to follow his suggestion, of keeping a spine, in which a removal of bone has occurred, stretched upon an inclined plane. In the early stages, where antiphlogistic and counter-irritant means are required, to avert or arrest the mischief, although the patient must be kept perfectly quiet in bed, yet who is there would *stretch* him on an inclined plane? Suppose that the disease has advanced, even to the destruction and removal of more or less of the bodies of the vertebræ, who is there, aware that this bone will not be reproduced by insisting upon a forcibly extended position, would frustrate the mode of cure that Nature herself adopts? namely, the falling together and anchylosing of the vertebræ, which bound the chasm above and below, thereby compensating for the loss of substance; but, by these very means, necessitating the angular projection. Suppose the patient is not seen until angular projection has been thus formed, who, by extension and compression, would seek to restore the uniformity of the spinal column, at the expense of risking the integrity of its canal?

In a concluding chapter, Mr. Hare gives us his ideas upon *pulmonary consumption:* want of space prevents our pursuing this subject; but, that his opinions are amply open to criticism, the reader will believe, when we inform him that the author considers the disease rarely if ever hereditary, but as originating in prior inflammatory attacks; that it is a more or less curable affection, while a most excellent preventive consists in three months' confinement to his apparatus, thereby expanding the chest, straightening the spine, and improving the general health!

The author has put forth his book rather ostentatiously, as the result of forty years' experience, given to the world as a kind of duty or legacy; yet, assuredly, of the suggestions it contains, most were previously (or at least suggestions of a similar nature) well known to those accustomed to treat spinal diseases mechanically, while some of them we consider as rather injurious. The book has been injudiciously *extended* from its legitimate size of a two or three shilling pamphlet to a ten shilling octavo, and its *tout-ensemble* impresses us with a fear, that the evil spirit of bookmaking has been somewhat officious in its production. We must not omit to mention that each division of the subject is illustrated by a case, in which the cure of the patient by these means is related; while there are plates representing the state of the patients before and after treatment was commenced.

